# Psychiatric disorders in adolescents living with HIV in Botswana

**DOI:** 10.1186/s12981-022-00490-z

**Published:** 2023-01-04

**Authors:** Anthony A. Olashore, Saeeda Paruk, Oluyemi O. Akanni, Bonginkosi Chiliza

**Affiliations:** 1 Department of Psychiatry, Nelson R Mandela School of Medicine, University of KwaZulu-Natal, Durban, South Africa; 2 Department of Psychiatry, Faculty of Medicine, University of Botswana, Gaborone, Botswana; 3 Department of Clinical Services, Federal Neuro-Psychiatric Hospital, Benin City, Nigeria

**Keywords:** Psychiatric disorders, Congenitally infected adolescents, Behaviourally infected HIV adolescents, Botswana

## Abstract

**Background:**

As children living with HIV transition from adolescence into adulthood, they face a considerable burden of psychiatric disorders (PDs) which may vary between the perinatally and behaviorally infected. The knowledge of the pattern of these PDs in relation to the varying needs of the adolescents living with HIV (ALWHIV) is unclear but necessary for maximizing their linkage to care and improving their quality of life in Botswana.

**Aim:**

To determine the pattern of PDs in ALWHIV in Botswana; to compare and explore the differences in the pattern and their associated factors between congenitally infected adolescents (CIAs) and behaviorally infected adolescents (BIAs).

**Methods:**

A cross-sectional survey of 622 ALWHIV (399 CIA and 223 BIA) with the Mini International Neuropsychiatric Interview-Kid Screen.

**Results:**

The participants' mean age (SD) was 17.71 (1.60) years, with more males (54%), of whom 52.9% had at least one PD, with depression (23.6%) and generalised anxiety disorder (18.0%) being the most prevalent. The externalising disorders were associated with being CIA (OR = 3.99; 95% CI:1.87–8.54), male gender (OR = 3.93; 95% CI:2.02–7.64), and a viral load of 400 and above copies (OR = 3.53; 95%CI:1.92–6.48). Internalising disorders were associated with being BIA (OR = 3.64; 95%; CI: 2.39–5.56), females (OR = 2.59; 95% CI:1.75–3.83), poor counselling (OR = 2.23; 95% CI: 1.42–3.51) and struggling to accept HIV status (OR = 1.73; 95% CI:1.14–2.62).

**Conclusions:**

Depression and anxiety disorders were the most prevalent PDs in ALWHIV, who differed in psychiatric presentations, the BIAs being more likely to present with internalizing disorders, while the CIAs had more externalizing disorders. Due to the varying needs of ALWHIV, individualized management plans that consider gender, mode of infection, and other psycho-social needs, should be further studied and encouraged.

**Supplementary Information:**

The online version contains supplementary material available at 10.1186/s12981-022-00490-z.

## Background

A better understanding of psychosocial and emotional problems in children and adolescents living with HIV is a priority [1], as they face a considerable burden of mental and behavioral disorders. Behavioral and externalizing disorders are more prevalent among HIV infected adolescents than those exposed but uninfected or HIV-negative [2, 3].

The pattern of psychiatric disorders among adolescents living with HIV (ALWHIV) varies considerably across geographical regions and depends on the psychological measures used [4]. An older review of published studies on the prevalence of Diagnostic and Statistics Manual (DSM-IV) psychiatric disorders among ALWHIV found rates of 29% for attention deficit hyperactivity disorder (ADHD), 24% for anxiety disorders, and 25% for depression [5]. However, more recent reviews and meta-analyses have shown a different pattern, with depression and anxiety being the most prevalent psychiatric disorder, while behavioral disorders, such as ADHD, were becoming less common [4, 6]. For example, in one recent metanalysis, depressive disorder accounted for 22% (95% CI:12–34) and anxiety for 26% (95%, CI:12–44), while ADHD prevalence was 6% (95% CI: 4–9) [4].

In Botswana, where more than 20% of the population lives with HIV, only one study reported a high Paediatric Symptom Checklist (PSC) score and treatment failure among 8–17 years children and adolescents with HIV [4, 7]. The authors reported psychosocial problems in approximately 31% of perinatally infected children and adolescents,  and also found an association between a PSC score of greater than 20 and virological failure [7]. While it is evident that ALWHIV in Botswana suffer from psychological distress, the pattern and associated factors are unclear, with the psychiatric services specific to them being is very rudimentary. Thus, this dearth of knowledge poses a significant challenge to formulating need-based treatments in Botswana and calls for more research.

As children with HIV transition from adolescence into adulthood, their needs evolve, especially across gender and mode of infection (MOI) [8], these being complicated by poverty, stigma, and sexual relationship issues. These emerging issues deserve a differentiated care approach to deliver tailored, appropriate services to ALWHIV [8, 9]. Currently, a priority area is the effect of the MOI on their management [10], with two major groups having been described. The behaviorally infected adolescent (BIA) group consists of those who contacted the infection horizontally, from person-to-person, via an exchange of contaminated material or unprotected sexual relations. The congenitally infected adolescent (CIA) group, or those who contracted it vertically, includes adolescents born with the infection. The differences between these two groups have become important, particularly in low- and middle-income countries (LMICs), where the meager available resources need to be optimized [4, 10].

As the CIAs have a longer duration of exposure to the previously mentioned HIV psychosocial ramifications, it could be assumed that they would be more affected and likely to present in different ways than the BIA, who have a shorter duration of illness. Contrary to this postulation, one study from South Africa found a difference in the psychological needs between the groups, with the BIAs having more psychological problems, such as depression, anxiety, and substance use, than the CIAs [10]. However, while this study has shed some light on this subject, there are still many gaps relating to other disorders and geographic areas.

We, therefore, aimed to determine the pattern of PDs in ALWHIV in Botswana; to compare and explore the difference in the pattern and their associated factors between the CIAs and BIAs. This information may assist in developing relevant plans for an equitable distribution of scarce services among ALWHIV.

## Materials and methods

### Study design and setting

The cross-sectional study was conducted among ALWHIV aged 12 to 19 years. It is a multisite study in Botswana, including the Gaborone district health management team (DHMT), Botswana Baylor Children's Clinical Centre of Excellence (BBCCCE), Mahalapye, and Lobatse DHMT.

### Sampling and procedure

The sample size was estimated using the formula for comparing proportions in two groups [11], with a minimum sample size of 221 being calculated. Five psychology graduates were employed as research assistants and trained on the research instruments, as well as how to approach the parents and participants on clinic days.

Due to the sample's nature and the rarity of some categories, such as the BIA, participants were recruited as they came to the clinic until the required sample size was achieved. Every eligible participant referred by the clinician was approached and briefed about the study on the clinic day, with those willing to participate in the study being screened and recruited. Inclusion criteria included ALWHIV aged 12–19 years, on antiretroviral treatment (ART) for a minimum of six months, able to communicate in Setswana or English, and willing to participate.

Eligible participants were interviewed privately, their autonomy and confidentiality assured, and their clinical records reviewed. All COVID-19 protocols were observed for all the data collected during the pandemic. Data were collected from January 2020 to December 2021 due to the disruptions caused by the COVID-19 restrictions.

### Measures

The socio-demographic and clinical questionnaires were designed based on the reviewed literature to obtain variables such as gender, age, level of education, ethnic group, frequency of clinic attendance, and viral load. The MOI was determined by participants' self-reports and parent reports and corroborated with patients' records. Participants' responses were removed from the analysis if the mode of infection could not be determined. The frequency of clinic attendance and viral load were also corroborated with the patient's records, albeit with their and their primary caregivers' consent. The variable on counselling by the health staff was frame as, ‘the health staff who attend to me, regularly counsel me and give me adequate supports regarding my treatments.’ The responses were (i) Disagree strongly (ii) Disagree a little iii) Neither agree or disagree (iv) Agree a little (v) Agree strongly. Also, the variable, acceptability of status, was generated from the subjective question on how they feel about their status. There were three main responses: ‘still struggling,’ ‘has accepted status,’ and ‘do not know my status.’ However, the last response was excluded from the analysis. A more detailed description of this has been reported in a previous manuscript [12].

Mini International Neuropsychiatric Interview-Kid Screen MINI-KID [13] was used to assess psychiatric disorders among the participants. It is a structured clinical diagnostic interview organized into different modules, each module consisting of two parts: the screening and the diagnostic/specifier. Nine modules that are common in the literature were used: depression, suicidality, post-traumatic stress disorder (PTSD), anxiety disorder, adjustment disorder, ADHD, conduct disorder, oppositional defiant disorder (ODD), and psychosis. It comprehensively assesses the existence of 24 ICD-10 and DSM-IV mental disorders, is interviewer-administered, and has been used in the various adolescent populations in Africa, including Botswana [3, 14] and ALWHIV in sub-Saharan Africa [3]. All the instruments were translated into Setswana (the predominant language in Botswana) using the back-translation procedure (translated to Setswana and back to English) by a panel of bilingual experts.

### Ethical considerations

Approval was obtained from the University of KwaZulu-Natal Biological Research Ethics Committee and the University of Botswana Research and Ethical Review Committee (UBIRB). Written informed consent was obtained from the participants who were old enough to consent, while the parents gave consent for those under 18 years who assented to participate.

### Data analysis

The data was analyzed with the Statistical Package for Social Sciences-Version 21 (SPSS for windows 21), with frequency tables being employed for descriptive statistics, such as the socio-demographic and clinical variables. Bar graphs were used to represent the prevalence of PDs, their distributions by gender, and mode of infection (MOI), using chi-square tests. Age was expressed as means and standard deviations, while categorical variables, such as gender, externalizing, and internalizing disorders, were presented in percentages. Bivariate tests were used to show the relationships between the PDs (externalizing and internalizing disorders) and all the categorical and continuous variables. All the variables, regardless of their p-value were entered into a logistic regression model to explore these relationships further with the outcomes. The outcome, externalizing disorder, was operationally defined as meeting the criteria for at least one of ADHD, ODD, and conduct disorder, while internalizing disorders were either depression or anxiety disorder. The level of statistical significance for all tests was set at p < 0.05.

## Results

### Socio-demographic characteristics

Of the 743 participants interviewed, only 622 (83%) had complete responses on the variable of interest and were entered into SPSS for analysis. Others were discarded due to incomplete response, difficulty in establishing the MOI, and not meeting the inclusion criteria such as age and HIV status.

Their mean age (SD) was 17.71 (1.60) years, there were more males (54%) than females, with 399 (64.1) being CIA and 223 (35.2%) BIA. The two group were not different in terms of gender (χ2 = 0.04; p = 0.84), and level of education (χ2 = 0.01; p = 0.932). However, the BIAs were significantly older than the CIA participants (t = − 6.08; p < 0.01). Significantly, more CIA were maternally orphaned (χ2 = 6.53; p = 0.011), while more BIA had lost their fathers (χ2` = 4.99; p = 0.026). Significantly, a higher proportion of the BIAs had difficulty accepting their HIV status (χ2 = 41.2; p < 0.01) (Table 1).

**Table 1:** The socio-demographic characteristics of ALWHIV by mode of infection

Characteristics	CIA N = 399 N (%)	BIA N = 223 N (%)	P-value
Mean age in years (SD)	17.42 (1.80)	18.22 (1.01)	**<** **0.01**
Gender			0.843
Male	218 (54.6)	120 (53.8)	
Female	181 (45.4)	103 (46.2)	
Religion*			0.961
Christianity	318 (79.9)	178 (80.2)	
African traditional religion	11 (2.8)	6 (2.7)	
No religion	61 (15.3)	32 (14.4)	
Others	8 (2.1)	6 (2.8)	
Frequency of religious participation*			0.198
Regular	81 (20.5)	55 (25.0)	
Rarely or never	314 (79.5)	165 (75.0)	
Type of caregiver			0.394
Single parent	253 (63.4)	149 (66.8)	
Both parent	146 (36.6)	74 (33.2)	
Highest level of Education*			0.122
Primary	15.4%	10.0%	
Secondary	45.3%	51.4%	
Post-secondary	39.2%	38.6%	
Paternal orphaned			**0.026**
Yes	76 (19.5)	61 (27.4)	
No	316 (79.2)	162 (72.6)	
Maternal orphaned			**0.011**
Yes	124 (31.1)	48 (21.1)	
No	275 (68.9)	175 (78.5)	
Double orphaned			0.458
Yes	76 (19.0)	48 (21.5)	
No	323 (81.0)	175 (78.5)	
Counselling by care providers			0.859
Poor	105 (26.4)	57 (25.8)	
Good	292 (73.6)	164 (74.2)	
Clinic attendance			
Poor	33 (8.3)	27 (12.2)	0.116
Good	364 (91.7)	194 (87.8)	
Feelings about status*			** < 0.01**
Difficulty in accepting status	85 (23.9)	109 (50.0)	
Has accepted status	271 (76.1)	109 (50.0)	
Viral load*			0.260
Below 400 copies	289 (75.5%)	151 (71.2%)	
400 copies and above	94 (24.5%)	61 (28.8%)	

Significant p values in bold. *Total number not equal to 399 or 223 due to missing value

### Prevalence and pattern of psychiatric disorders among ALWHIV

**Fig. 1:**
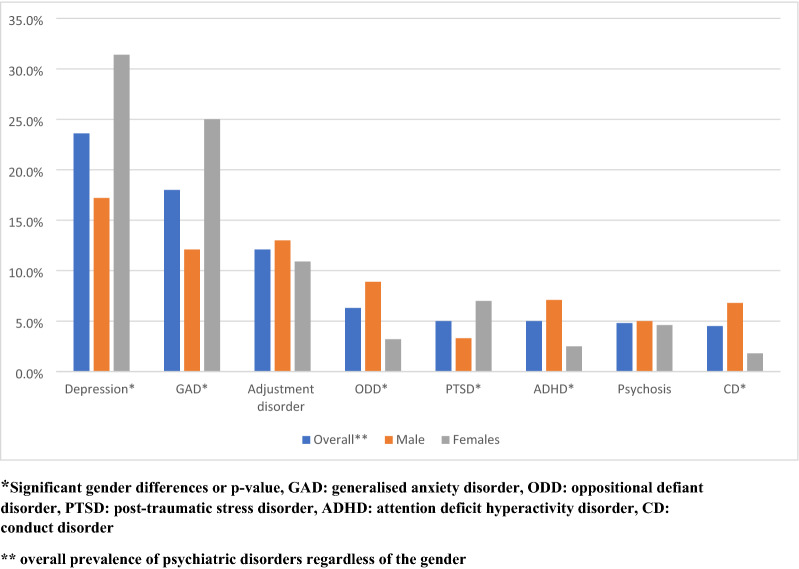
Pattern of psychiatric disorders by gender in ALWHIV

More than half (52.9%) of the participants had one psychiatric disorder, while 19.1% had more than one PDs, with depression (23.6%) and generalised anxiety disorder (18.0%) being the most prevalent. Conduct disorder and psychosis were the least common psychiatric disorders, accounting for 4.5% and 4.8%, respectively (Fig. 1). Internalising disorders, which included anxiety and depression, accounted for 37.1% while the externalising disorders, such as ADHD, ODD and conduct disorder, accounted for 11.6%. Depression (χ2 = 17.2; p < 0.01), generalised anxiety disorder (χ2 = 17.3; p < 0.01) and PTSD (χ2 = 4.68; p = 0.031) were significantly more common among the females, while ADHD (χ2 = 7.00; p = 0.008), conduct disorder (χ2 = 9.13; p = 0.003) and ODD (χ2 = 8.55; P = 0.003) were higher among males (Fig. 1).

**Fig. 2:**
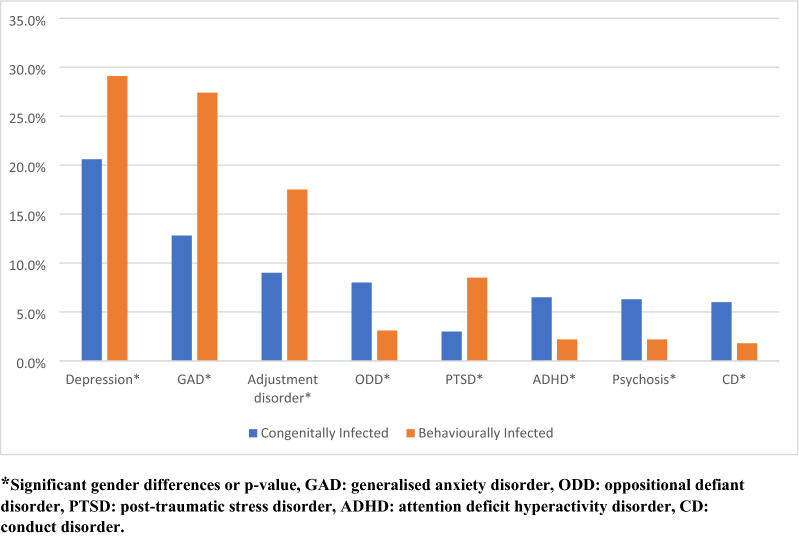
Pattern of psychiatric disorders by mode of infection in ALWHIV

Figure 2 shows the prevalence of psychiatric disorders by MOI, with the BIA having significantly more internalising disorders, such as depression (χ2 = 10.53 *p* = 0.016,) and anxiety (χ2 = 20.6, p < 0.01). They were also more likely to have PTSD (χ2 = 9.18, p = 0.002) and adjustment disorders (χ2 = 9.67, *p* = 0.002) than the CIAs, who had more externalising disorders, such as ADHD (χ2 = 10.9, p < 0.01), and psychosis (χ2 = 5.05, p = 0.025).

### The predictors of externalizing and internalizing psychiatric disorders in the ALWHIV

The bivariate tests indicating the relationship between the participants’ characteristics and the outcome is shown in Additional file 1: Tables S1 and S2. All the variables, regardless of their p-value were entered into the logistic regression model, with Table 2 showing the factors associated with externalizing psychiatric disorders. Male participants (OR = 3.93; 95% CI:2.02–7.64), those who had a viral load of 400 and more copies (OR = 3.53; 95%CI:1.92–6.48), and the CIAs (OR = 3.99; 95% CI:1.87–8.54) were more likely to have externalising disorders. Conversely, female participants (OR = 2.59; 95% CI: 1.75–3.83) and the BIAs (OR = 3.64; 95%; CI: 2.39–5.56), were more than two times more likely to present with internalizing disorders (Table 3). In addition, those who complained of poor counselling from health staff (OR = 2.23; 95% CI: 1.42–3.51) and felt bad or had yet to accept their HIV status (OR = 1.73; 95% CI:1.14–2.62) were more likely to have internalizing disorders (Table 3). An ad-hoc analysis was conducted to explore the relationship between externalizing disorders and viral load while controlling for the effect of MOI. In the CIAs, having a viral load of 400 and above was associated with externalizing disorder (OR = 3.85; 95% CI: 2.16–6.87), while this relationship was not significant in the BIAs (OR 0.61; 95%CI: 0.13–2.94).

**Table 2:** Model showing the predictors of externalizing disorders in ALWHIV

Characteristics	B	S. E	Wald	*p*	OR	95% CI	
						Lower	Upper
Gender							
Male	1.37	0.34	16.3	** < 0.01**	3.93	2.02	7.64
Age							
Older age	0.07	0.11	0.45	0.503	1.08	0.87	1.33
Counselling from health staff							
Poor	0.44	0.34	1.73	0.189	1.55	0.81	2.98
Paternally orphaned							
Yes	− 0.98	0.61	2.63	0.105	0.38	0.12	1.23
Maternally orphaned							
Yes	− 0.56	0.49	1.30	0.254	0.57	0.22	1.49
Viral load							
Below 400 copies	1.26	0.31	16.4	** < 0.01**	3.53	1.92	6.48
Mode of infection							
Congenitally	1.39	0.39	12.8	** < 0.01**	3.99	1.87	8.54
Feelings about status							
Struggling to accept status	− 0.20	0.35	0.33	0.565	0.82	0.41	1.62
Perceived support from family							
Good	− 0.12	0.30	0.16	0.686	0.89	.50	1.58

Significant p values in bold

## Discussion

Our study described the presence of PDs in ALWHIV, compared their pattern, and explored their associated factors between CIAs and BIAs. The most common PDs in ALWHIV were internalizing disorders, such as depression and anxiety, with the BIA being more likely to have internalizing disorders while the CIAs had more externalizing disorders.

The BIAs were significantly older than the CIAs, as also documented in a previous study with a similar design [10]. As the BIAs were possibly older adolescents than the CIAs, it is reasonable that their psychosocial needs would be different. In addition, the CIAs were observed to have been more maternally orphaned than their BIAs infected counterpart, which may be related to high maternal mortality before the widespread use of ART to prevent mother-to-child transmission (PMTCT)[15]. It should be noted that disruption of mothering may have far-reaching effects on these individuals' treatment outcomes and quality of life [10], especially in Botswana, where pediatric care mainly lies with single mothers [16]. As suggested by a previous study that observed similar findings [10], providing bereavement support to the affected adolescents may improve their attitude and response to HIV treatment.

**Table 3:** Model showing the predictors of internalizing disorders in ALWHIV

Characteristics	B	S. E	Wald	*p*	OR	95% CI	
						**Lower**	**Upper**
Gender							
Female	0.95	0.20	22.7	** < 0.01**	2.59	1.75	3.83
Age							
Older age	0.08	0.08	1.18	0.278	1.09	0.94	1.26
Counselling from health staff							
Poor	0.80	0.23	12.0	**0.001**	2.23	1.42	3.51
Paternally orphaned							
Yes	− 0.36	0.35	1.07	0.300	0.70	0.35	1.38
Maternally orphaned							
Yes	0.30	0.33	0.83	0.363	1.35	0.71	2.60
Viral load							
Below 400 copies	0.37	0.22	2.77	0.096	1.45	0.94	2.24
Mode of infection							
Behaviourally	1.29	0.22	36.0	** < 0.01**	3.64	2.39	5.56
Feelings about status							
Struggling to accept status	0.55	0.21	6.70	**0.010**	1.73	1.14	2.62
Perceived support from family							
Poor	0.36	0.20	3.24	0.072	0.70	0.47	1.03

Significant p-values in bold

The pattern of PDs observed in our cohort followed the widely documented trend in sub-Saharan Africa [4, 6], where depression and anxiety were the most commonly presented mental health conditions in ALWHIV. It is also worth noting that, contrary to a report before the era of ART, which noted that externalizing disorders dominated [5], the current study found that they were observed to be less prominent than internalizing disorders, in keeping with more recent reports [4, 6]. Thus, this supports the recent position [4, 6] that ALWHIV are more likely to suffer from disorders relating to stigma, poverty and orphanhood, such as depression, than the externalizing symptom, which may be a direct consequence of the viral exposure.

Regarding the pattern of psychiatric disorders by MOI, our findings replicated a previous report [10], where anxiety and depression were more prevalent among the BIAs than the CIAs. In addition [10], the present study found that the CIAs who had lived with the infection for an extended period had disorders such as psychosis, ADHD and conduct disorder, although the rate was lower than in the pre-ARV treatment era [5].

While sub-classifying the disorders into externalizing and internalizing disorders, we sought to explore the predictors of PDs among ALWHIV using a multivariate logistic regression model. Apart from gender and MOI, factors associated with externalizing disorders were different from those with internalizing disorders, with males being more likely to present with the former while females were more likely to have the latter. Studies among seropositive [10, 17, 18] and seronegative adolescents [19, 20] have consistently reported a similar gender difference in the prevalence of psychiatric disorders, with internalizing disorders being commoner among females [4, 17] and externalizing disorders among males [19, 20]. Regardless of the status, males may have more difficulties admitting that they have psychological problems and tend to mask their emotions or cope in a maladaptive way, such as using drugs or acting out externalizing symptoms. Conversely, females appear to be more emotionally sensitive, which may be related to their socially defined roles, hence the tendency to engage in self-absorption or ruminative self-focus and self-directed aggression as a way of coping with stress [21, 22].

Our study revealed the association between MOI and both externalizing and internalizing psychiatric disorders. While the BIAs were three times more likely to present with internalizing disorders, the CIAs were about four times more likely to present with externalizing disorders.

The present finding replicated the report by Sherr and colleagues [10], who also reported a higher prevalence of internalizing disorders of anxiety and depression among the BIAs than the CIAs. This finding further buttresses the earlier claim that adolescents who acquire the infection have different needs from those born with it. Although the present study could not determine a causal relationship, the types of psychological disorders suggests that the BIAs suffer from adjusting to a new lifestyle, medication, and stigma, and may therefore display more maladaptive coping. Those who struggle to accept their status were, therefore, not unexpectedly almost twice as likely to present with an internalizing disorder. The BIAs were significantly more likely to struggle with their HIV status acceptance than the CIAs. Kraaij and colleagues [23] have suggested that ALWHIV have increased vulnerability to negative emotions, such as catastrophizing, negative rumination, self-blaming, and abnormal emotional responses to adverse life events. Another study contended that shame was one of the reasons for poor adjustment to HIV status in infected individuals [24].

Although we did not assess or compare stigma in our cohort, a South African study reported that the BIAs are more likely to report shame and stigma than their CIA counterpart [10]. Moreover, there was no relationship between struggling with status acceptance and internalizing disorders in the CIA subgroup. Therefore, it is reasonable to suggest that the development of internalizing disorders by the BIAs was partly related to difficulty in accepting their new status. As suggested by Sherr and colleagues [10], this may have serious consequences for their retention in care, treatment outcome and general quality of life. This further strengthens the need to conduct counseling regarding coping and acceptance in the care of ALWHIV.

Internalizing disorders were also associated with a poor perception of support and counseling from the care provider, which was common in both groups. Perhaps the low satisfaction stemmed from the lack of an individualised approach to care in African settings and needs a review, as studies have shown the need for a tailored approach to health care delivery in this cohort to optimize linkage and retention in care [9, 10]. This approach includes need-based counseling, as using the same strategy for the two groups may be counter-productive. The BIAs, who may be older, naïve to treatment, more sensitive to stigma and more likely to be exposed to sexual relations, may require a different approach from the CIAs, who are more experienced with treatment and less likely to have been sexually exposed. For example, in the developing countries, the uninfected society hold a stigmatizing belief that people living with HIV (PLWHIV) possess moral blemishes such as intravenous drug use, risky sexual behavior, and homosexuality and show a negative attitude toward them [25–27]. Likewise, those infected live with self-blame and psychological responses associated with the knowledge that they have violated social mores and are sick because of the consequence of their lifestyle [25].

The CIAs were almost four times more likely to present with externalizing disorders than the BIAs, and while no study has compared this between the two groups, to the best of our knowledge, authors have consistently reported the presence of externalizing among the CIAs [4, 18, 28]. Externalizing disorders, such as ADHD, may occur partly due to the effect of HIV on the brain, with studies having shown a drastic reduction in the prevalence of ADHD post ART era compared to pre-ARV [4–6]. Additionally, authors have suggested a correlation between externalizing disorder and a prolonged period of poor ART adherence in ALWHIV [29] with poor neuropsychological functioning being found to be worse in children with higher viral loads [30]. Persistent or chronic exposure to viral product expression has been linked to increased glial cell abnormalities. Glial dysfunctions were shown lead to abnormalities in glutamatergic systems, neuronal migration, and metabolic supports, resulting in neurodevelopmental damage and disorders such as hyperactivity [31]. In support of this claim, we found a strong association between externalizing disorders and viral load above 400 copies. Although there was no difference between the two groups in the present study, an ad-hoc analysis between viral load and externalizing disorder revealed that a significant association only existed in the CIA group, which is expected, as they had lived with the virus longer than the BIAs. Should both groups have poorly suppressed viral loads, as we observed, the prolonged viral exposure could have affected the CIAs more than the BIAs. Therefore, our findings further buttress the need for more individualized or tailored services in order to optimize the meager available resources in Botswana [10].

We observed some limitations that may have affected the results. First, we cannot ascertain the temporal relationship between the dependent and independent variables due to the cross-sectional nature of the study. Secondly, there is a possibility that we may have excluded some BIAs whose MOI could not be verified due to poor record-keeping and the absence of caregivers to corroborate their status. The self-report nature of some of the questionnaires, particularly the socio-demographic part, and their subjectivity, may also have introduced some reporting bias. For example, a single item question on the acceptability of status may not have explored sufficiently the various dimension of the construct; it would be more appropriate to apply a standardized scale, if available for acceptable validity. The possible effect of overfitting in our model build-up is noted and suggests a careful interpretation of our findings. Finally, we are not oblivious to the possible effect of COVID-19 on our results, as some samples were taken when restrictions on movement were imposed, possibly affecting people’s mental status. For example, the fear of getting infected could have affected the rate of disorders, such as generalized anxiety disorder (GAD). However, the diagnostic criteria require six months of symptoms to diagnose GAD and many others, such as psychosis and ADHD. Nonetheless, we advise a cautious interpretation of our results as this diagnostic rule does not apply to all the disorders.

## Conclusions

The prevalence of psychiatric disorders was high, with ALWHIV presenting with more internalizing than externalizing disorders, as was reported during the pre-ARV treatment era. Depression and anxiety disorders were the most common internalizing disorders, these occurring more among females and BIAs, while ODD and ADHD were the most common externalizing disorders, affecting the males and CIAs. Another factor that predicted internalizing disorder was difficulty accepting HIV status, which was also related to the BIAs, while a viral load of 400 and above predicted externalizing disorder among those in the CIA group.

These disparities in their psychosocial needs and the association between lack of satisfaction with services and internalizing disorders supports the need to rethink the generally practiced ‘broad-brush approach’ to health care and prevention in ALWHIV in many African settings [10, 32]. Also, more research should focus on the benefits and proper implementation of individualized management plans that consider adolescents' peculiar factors, such as gender, MOI and other psychosocial needs since this is not being currently done.

## Supplementary Information

**Additional file 1: Table S1.** Relationship between the characteristics of ALWHIV and Externalising disorders. **Table S2.** Relationship between the characteristics of ALWHIV and Internalising disorders.

## Data Availability

The datasets used and analyzed during the current study are available from the corresponding author on reasonable request.

## Abbreviations

ADHD: Attention deficit hyperactivity disorder
ALWHIV: Adolescents living with HIV
ART: Antiretroviral Therapy
BIA: Behaviourally infected
CIA: Congenitally infected adolescents
DSM-5: Diagnostic and Statistical Manual of Mental Disorders, fifth edition (DSM-5)
IRB: Independent Review Board
LMICs: Low to middle-income countries
MINI-KID: Mini International Neuropsychiatric Interview for Children and Adolescents
MOI: Mode of Infection
ODD: Oppositional defiant disorder
PDs: Psychiatric disorders
PTSD: Posttraumatic stress disorder
