# Supplementary material for: Psychiatric disorders in adolescents living with HIV in Botswana
Source: AIDS Res Ther. 2023 Jan 4;20:2. doi: 10.1186/s12981-022-00490-z (PMC9812345; doi:10.1186/s12981-022-00490-z)
Supplement: Supplementary file 1 — Additional file 1: Table S1. Relationship between the characteristics of ALWHIV and Externalising disorders. Table S2. Relationship between the characteristics of ALWHIV and Internalising disorders. [file 12981_2022_490_MOESM1_ESM.docx]

**Table 4: Relationship between the characteristics of ALWHIV and Externalising disorders**

| **Characteristics** | **B** | **S. E** | **Wald** | ***p*** | **OR** | **95% CI.** | |
| --- | --- | --- | --- | --- | --- | --- | --- |
|  |  |  |  |  |  | **Lower** | **Upper** |
| **Gender** | 0.95 | 0.28 | 11.5 | **0.001** | 2.59 | 1.50 | 4.50 |
| Male | Ref |  |  |  |  |  |  |
| **Age** | -0.11 | 0.07 | 2.42 | 0.120 | 0.89 | 0.78 | 1.03 |
| Older age | Ref |  |  |  |  |  |  |
| **Counselling from health staff** | 0.03 | 0.285 | 0.01 | 0.911 | 1.03 | 0.59 | 1.80 |
| Poor | Ref |  |  |  |  |  |  |
| **Paternally orphaned** | 1.26 | 0.42 | 8.27 | **0.004** | 3.52 | 1.49 | 8.31 |
| Yes | Ref |  |  |  |  |  |  |
| **Maternally orphaned** | .831 | .341 | 5.946 | **.015** | 2.295 | 1.177 | 4.475 |
| Yes | Ref |  |  |  |  |  |  |
| **Viral load** | -0.97 | .26 | 13.684 | **<0.01** | 0.38 | 0.23 | .633 |
| Below 400 copies | Ref |  |  |  |  |  |  |
| **Mode of infection** | 1.37 | 0.35 | 15.1 | **<0.01** | 3.92 | 1.97 | 7.81 |
| Congenitally | Ref |  |  |  |  |  |  |
| **Feelings about status** | 0.45 | 0.30 | 2.20 | 0.138 | 1.57 | 0.87 | 2.82 |
| Struggling to accept status | Ref |  |  |  |  |  |  |
| **Perceived support from family** | -0.23 | 0.25 | 0.80 | 0.372 | 0.70 | 0.487 | 1.31 |
| Good | Ref |  |  |  |  |  |  |

**Table 5: Relationship between the characteristics of ALWHIV and disorders Internalising**

| **Characteristics** | **B** | **S. E** | **Wald** | ***p*** | **OR** | **95% CI.** | |
| --- | --- | --- | --- | --- | --- | --- | --- |
|  |  |  |  |  |  | **Lower** | **Upper** |
| **Gender** | 0.86 | 0.17 | 25.4 | **<0.01** | 2.35 | 1.69 | 3.28 |
| Females | Ref |  |  |  |  |  |  |
| **Age** | 0.12 | 0.05 | 4.69 | **0.030** | 1.12 | 1.01 | 1.25 |
| Older age | Ref |  |  |  |  |  |  |
| **Counselling from health staff** | 0.45 | 0.19 | 5.74 | **0.017** | 1.56 | 1.09 | 2.25 |
| Poor | Ref |  |  |  |  |  |  |
| **Paternally orphaned** | 0.13 | 0.10 | 0.41 | 0.523 | 1.14 | 0.77 | 1.68 |
| Yes | Ref |  |  |  |  |  |  |
| **Maternally orphaned** | -0.17 | 0.19 | 0.82 | 0.366 | 0.84 | 0.58 | 1.22 |
| Yes | Ref |  |  |  |  |  |  |
| **Viral load** | -0.44 | 0.19 | 5.24 | **0.022** | 0.65 | 0.45 | 0.94 |
| Below 400 copies | Ref |  |  |  |  |  |  |
| **Mode of infection** | 1.29 | 0.18 | 53.5 | **<0.01** | 3.64 | 2.57 | 5.14 |
| Behavioural | Ref |  |  |  |  |  |  |
| **Feelings about status** | 0.85 | 0.18 | 21.9 | **<0.01** | 2.33 | 1.64 | 3.33 |
| Struggling to accept status | Ref |  |  |  |  |  |  |
| **Perceived support from family** | -0.28 | 0.17 | 2.82 | 0.093 | 0.76 | 0.54 | 1.05 |
| Good | Ref |  |  |  |  |  |  |
